# Periplasmic Flagellar Export Apparatus Protein, FliH, Is Involved in Post-Transcriptional Regulation of FlaB, Motility and Virulence of the Relapsing Fever Spirochete *Borrelia hermsii*

**DOI:** 10.1371/journal.pone.0072550

**Published:** 2013-08-29

**Authors:** Cyril Guyard, Sandra J. Raffel, Merry E. Schrumpf, Eric Dahlstrom, Daniel Sturdevant, Stacy M. Ricklefs, Craig Martens, Stanley F. Hayes, Elizabeth R. Fischer, Bryan T. Hansen, Stephen F. Porcella, Tom G. Schwan

**Affiliations:** 1 Public Health Ontario, Toronto, Ontario, Canada; 2 University of Toronto, Toronto, Ontario, Canada; 3 Mount Sinai Hospital, Toronto, Ontario, Canada; 4 Laboratory of Zoonotic Pathogens, Rocky Mountain Laboratories, National Institute of Allergy and Infectious Diseases, National Institutes of Health, Hamilton, Montana, United States of America; 5 Research Technologies Section, Research Technologies Branch, Rocky Mountain Laboratories, National Institute of Allergy and Infectious Diseases, National Institutes of Health, Hamilton, Montana, United States of America; University of Kentucky College of Medicine, United States of America

## Abstract

Spirochetes are bacteria characterized in part by rotating periplasmic flagella that impart their helical or flat-wave morphology and motility. While most other bacteria rely on a transcriptional cascade to regulate the expression of motility genes, spirochetes employ post-transcriptional mechanism(s) that are only partially known. In the present study, we characterize a spontaneous non-motile mutant of the relapsing fever spirochete *Borrelia hermsii* that was straight, non-motile and deficient in periplasmic flagella. We used next generation DNA sequencing of the mutant’s genome, which when compared to the wild-type genome identified a 142 bp deletion in the chromosomal gene encoding the flagellar export apparatus protein FliH. Immunoblot and transcription analyses showed that the mutant phenotype was linked to the posttranscriptional deficiency in the synthesis of the major periplasmic flagellar filament core protein FlaB. Despite the lack of FlaB, the amount of FlaA produced by the *fliH* mutant was similar to the wild-type level. The turnover of the residual pool of FlaB produced by the *fliH* mutant was comparable to the wild-type spirochete. The non-motile mutant was not infectious in mice and its inoculation did not induce an antibody response. Trans-complementation of the mutant with an intact *fliH* gene restored the synthesis of FlaB, a normal morphology, motility and infectivity in mice. Therefore, we propose that the flagellar export apparatus protein regulates motility of *B. hermsii* at the post-transcriptional level by influencing the synthesis of FlaB.

## Introduction

Relapsing fever is a vector-borne illness caused by spirochetes of the *Borrelia* species. In North America, *Borrelia hermsii* and *Borrelia turicatae* are the primary aetiologic agents of human tick-borne relapsing fever. During their enzootic cycles, these two species of spirochetes alternate between mammals and tick species of the genus *Ornithodoros* [1], [2]. The properties that allow relapsing fever spirochetes to disseminate and invade their hosts and vector still need to be explored.

Spirochetes are long, thin, motile, helical or flat-wave bacteria that are unique among the prokaryotes by having flagella with axial filaments confined to an internal periplasmic space between the inner and outer membranes [3]. Many vector-borne spirochetes are pathogenic in humans. Infections occur when ticks feed, allowing for the transmission of spirochetes in tick saliva or coxal fluid. A common event for all borreliae that have been studied in their vector is their penetration of the arthropod’s midgut wall. This dissemination by borreliae out of the tick midgut following their ingestion with blood is a prerequisite for their subsequent transmission. The role of motility for spirochetes to escape specifically from the midgut lumen of ticks is not known, however, other species of pathogenic bacteria that lack motility are able to disseminate and infect salivary glands for their transmission via saliva [4].

The flex motility of spirochetes is produced by the periplasmic flagella, which share a common internal structure with the external flagella of other bacteria [5], [6], [7]. Periplasmic flagella are composed of a motor (basal body), hook, and filament [8]. In *B.* *burgdorferi*, periplasmic flagellar filaments comprise a major polymerized protein, FlaB and a minor protein, FlaA [8], [9]. *B. burgdorferi* contains between 7–11 periplasmic flagella that are attached near each pole of the cell cylinder [10]. In the relapsing fever spirochete, *Borrelia recurrentis,* the number of periplasmic flagella is between 8 to10 [11].

To recognize, unfold and translocate flagellar proteins, flagellated bacteria employ the flagellar type III protein export apparatus, which consists of six integral membrane proteins (FlhA, FlhB, FliO, FliP, FliQ and FliR) and three soluble proteins (FliH, FliI, FliJ) [12], [13]. Recent cryo-electron tomogram analyses of spirochetes suggest that an export apparatus with a similar structure is present among *Borrelia* species [8], [14].

In bacteria, expression of motility genes is generally under the control of the transcription factor σ-28. In contrast, the *flaB* gene of *B. burgdorferi* is constitutively transcribed through the control of the housekeeping transcription factor σ-70 [15], [16]. Gene inactivation showed that the FlaB protein was essential for motility and the flat wave shape of *B. burgdorferi* [17].

For some pathogenic bacterial species, flagella may contribute to their virulence [18], [19], [20]. Little is known about the contribution that periplasmic flagella make to the virulence of pathogenic spirochetes and very few spirochetes lacking periplasmic flagella have been described. A non-motile mutant of *Treponema denticola* was unable to penetrate the epithelial tissue layer *in vitro* [21]. A flagella-less, straight, non-motile, spontaneous mutant of *B. burgdorferi* with an intact *flaB* gene was cloned from a population of spirochetes that had already lost infectivity in mammals [22]. This non-motile mutant adhered equally well to mammalian tissue culture cells when compared to wild-type spirochetes, but was less able to penetrate human umbilical vein endothelial cell monolayers. Furthermore, virulence of *Brachyspira hyodysenteria* mutants with impaired motility was reduced in mice and pigs [23], [24]. More recently, the inactivation of a putative flagellar motor switch protein FliG1 was shown to block *B. burgdorferi* infectivity [25]. In *Leptospira interrogans,* a *flaA2* transposon mutant was not infectious in animal models of acute infection [26]. Furthermore, a site directed *flaB* motility mutant of *B. burgdorferi* was able to survive in ticks that were artificially infected by immersion but was non-transmissible by tick-bite and non-infectious in mice [27].

In this study, we isolated a non-motile mutant of the relapsing fever spirochete *B. hermsii* that originated from spirochetes cultured from the blood of a human patient acutely ill with relapsing fever. Here, we characterize this mutant and identify a unique mutation in BH0289 (*fliH*) using whole-genome sequencing analysis. We demonstrate that the deletion of BH0289 reduced the synthesis of the periplasmic flagella protein FlaB. Moreover, we analyzed the effect of the *bh0289* deletion on infectivity in mice. Our data demonstrate the role of FliH in FlaB post-transcriptional regulation, motility and pathogenicity of the spirochete *B. hermsii*.

## Results

### Identification of the Non-motile Mutant Spirochete

**Figure 1:**
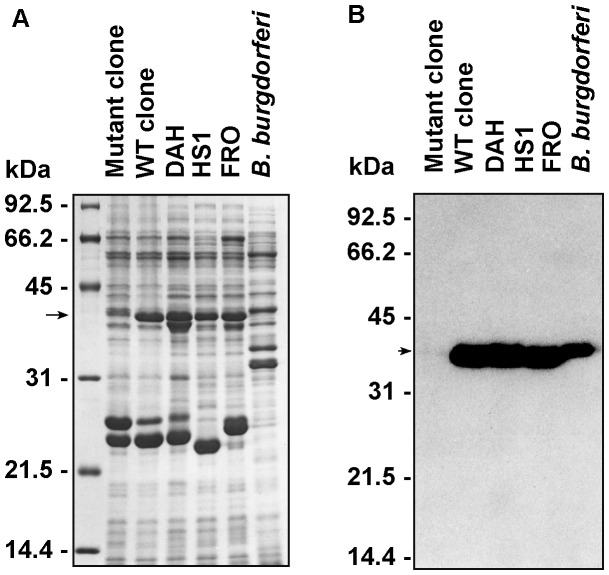
Protein and immunoblot analyses of flagella deficient *B. hermsii* compared to other isolates of *B. hermsii and B. burgdorferi*. (A–B) SDS-PAGE, Coomassie blue stain and immunoblots with an anti-FlaB monoclonal antibody H9724. Whole-cell lysates include the *B. hermsii* non-motile mutant (Mutant clone), wild type clone (WT clone), the parental strain (DAH), two additional *B. hermsii* isolates (FRO and HS1), and *B. burgdorferi* B-31. The non-motile mutant is deficient in the amount of the 39-kDa protein FlaB (arrow) (A). The anti-FlaB monoclonal antibody confirms the paucity of FlaB in the lysate of the mutant (arrow) (B). Molecular mass standards are shown on the left in kDa.

The original isolate of *B. hermsii* DAH came from a patient acutely ill during a relapse phase consistent with tick-borne relapsing fever. When the isolate was cloned by limiting dilution, a non-motile mutant was discovered in one of the wells. DAH, a wild-type clone (WT), and the non-motile mutant clone had protein profiles similar to other *B. hermsii* strains HS1 and FRO, except that the non-motile mutant appeared to be deficient in the synthesis of a 39 kDa protein (Fig. 1A). The growth rates of WT and the non-motile mutant were similar to that of the original isolate in BSK-II medium (data not shown), although the mutant cells had a tendency to clump and aggregate to themselves. Based on its estimated molecular mass and antigenicity, we speculated that the deficient protein observed in the mutant clone was FlaB. To verify this hypothesis, the synthesis of FlaB by mutant clone, WT, DAH and HS1 was examined by immunoblot analysis with the monoclonal anti-FlaB antibody H9724 (Fig. 1B). H9724 strongly reacted with FlaB of WT, DAH and HS1, confirming their identity as *Borrelia* species. Only weak reactivity was observed with the non-motile mutant, which suggested that the phenotype was linked to a deficiency in the synthesis of FlaB.

### Morphology of the Non-motile Spirochete

**Figure 2:**
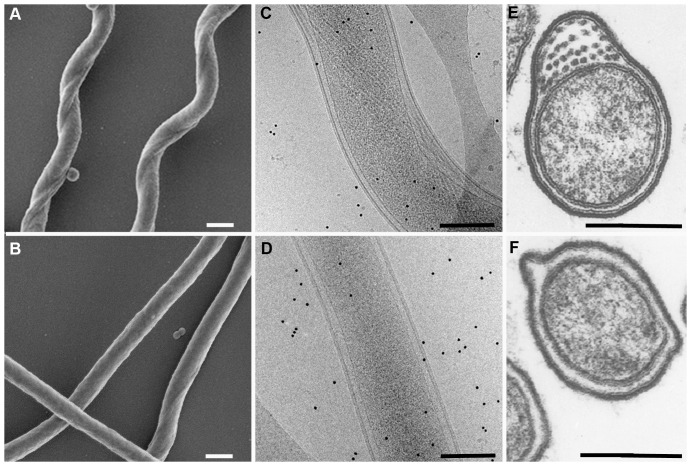
Electron microscopic (EM) analyses of parental wild-type *B. hermsii* and non-motile mutant. (A–F) The morphology of the mutant is strikingly different than the wild-type *B. hermsii*. By Scanning EM, the wild-type cells show the typical flat-wave morphology (A) compared to the straight shape of the non-motile mutant (B). By Cryo-EM, the wild-type spirochetes show the normal shape and numerous periplasmic flagella (C) compared to the mutant that is straight with few or no periplasmic flagella (D). Cross-sections viewed by transmission EM also show numerous flagella in the periplasmic space of the parental wild-type spirochetes (E) compared to none in the example of the non-motile mutant of *B. hermsii* shown here (F). All scale bars represent 0.2 µm.

The gross morphology of the mutant was strikingly different than wild-type spirochetes (Fig. 2). The mutant spirochetes were straight and deficient in the number of periplasmic flagellar axial filaments (Fig. 2B, D, F), whereas the wild-type spirochetes displayed the typical flat-wave morphology and numerous filaments (Fig. 2A, C, E). Samples of spirochetes were examined by different methods to assess the deficiency in the number of axial filaments in the mutant cells. One hundred mutant spirochetes were negative stained and examined by electron microscopy. Nearly half (48%) of these bacteria contained no visible axial filaments, while the remainder of the spirochetes had only 1 to 4 filaments per cell. Wild-type cells had the typical flat-wave morphology and contained 30–32 axial filaments (examples not shown). Spirochetes were also examined by Cryo-electron microscopy (examples in Fig. 2C, D). Evidence of flagella was observed in over 50 wild-type spirochetes examined, whereas little or no evidence of flagella were apparent in the mutant cells. Transmission electron microscopy was also used to view cross-sections of mutant and wild-type spirochetes. In this small sample, the mutant spirochetes had a clearly defined periplasmic space with either no or 1–4 filaments. In wild-type cells, the axial filaments numbered 27–32 when sampled in the middle of the spirochete where the filaments that arise from both poles of the bacterium overlap (examples in Fig. 2 E, F).

**Figure 3:**
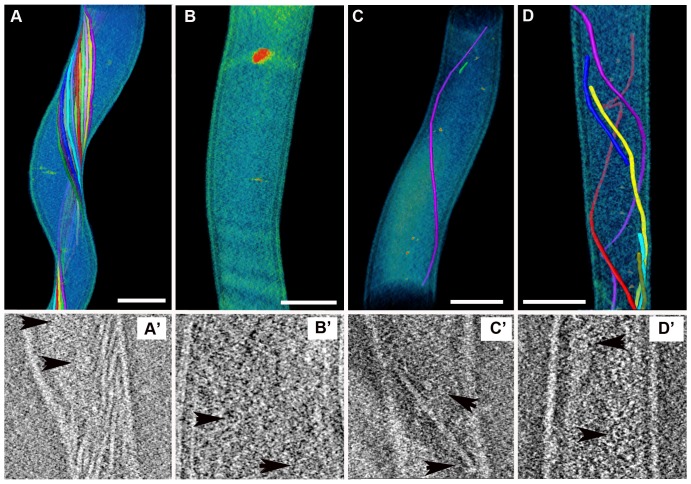
Electron microscope analysis of intact *B. hermsii* cells for the presence of periplasmic flagella. (A–D) 3-dimensional tomographic renderings of *B. hermsii* wild-type and mutant cells. The wild-type cell has numerous intact axial filaments each delineated by different colors (A), while the three mutant cells show either no (B), one intact (C), or several irregular and truncated filaments. (D). Ortho-slice views (A′–D′) taken from the same respective samples show the presence of basal bodies (black arrowheads) including the mutant cell lacking axial filaments (B & B′). The scale bar represents 0.2 µm.

Three-dimensional tomographic renderings were also constructed to better assess the structure of the periplasmic flagella in the mutant spirochetes (Fig. 3). Analysis of 53 mutant cells showed that 43% of these spirochetes had no visible axial filaments. The majority of the remaining cells (43%) contained only 1–3 axial filaments while 14% of the cells had 4 or 5 of these structures. The wild-type spirochetes had dense bands of periplasmic flagellar axial filaments wrapping around the cell cylinder (Fig. 3A). Again, these structures were either absent or few in number within the mutant spirochetes (Fig. 3B, C, D). Some mutant cells contained full-length (Fig. 3C) or truncated axial filaments of the flagellar apparatus that were also bent and grouped erratically (Fig. 3D). From these renderings, ortho-slice views through the same cells showed that some mutant cells without axial filaments did have one or more flagella basal bodies (Fig. 3B′) or basal bodies with truncated axial filaments (Fig. 3C′ and 3D′). Together, these multiple approaches to examine the morphology of the spirochetes showed the *B. hermsii* mutant spirochetes were either straight or slightly flat-wave in their shape, had either no intact periplasmic flagella or a greatly reduced number, some cells contained truncated axial filaments, and the absence of axial filaments did not preclude the cells from having flagellar basal bodies.

### Genetic Analysis of the Mutant Spirochete

**Figure 4:**
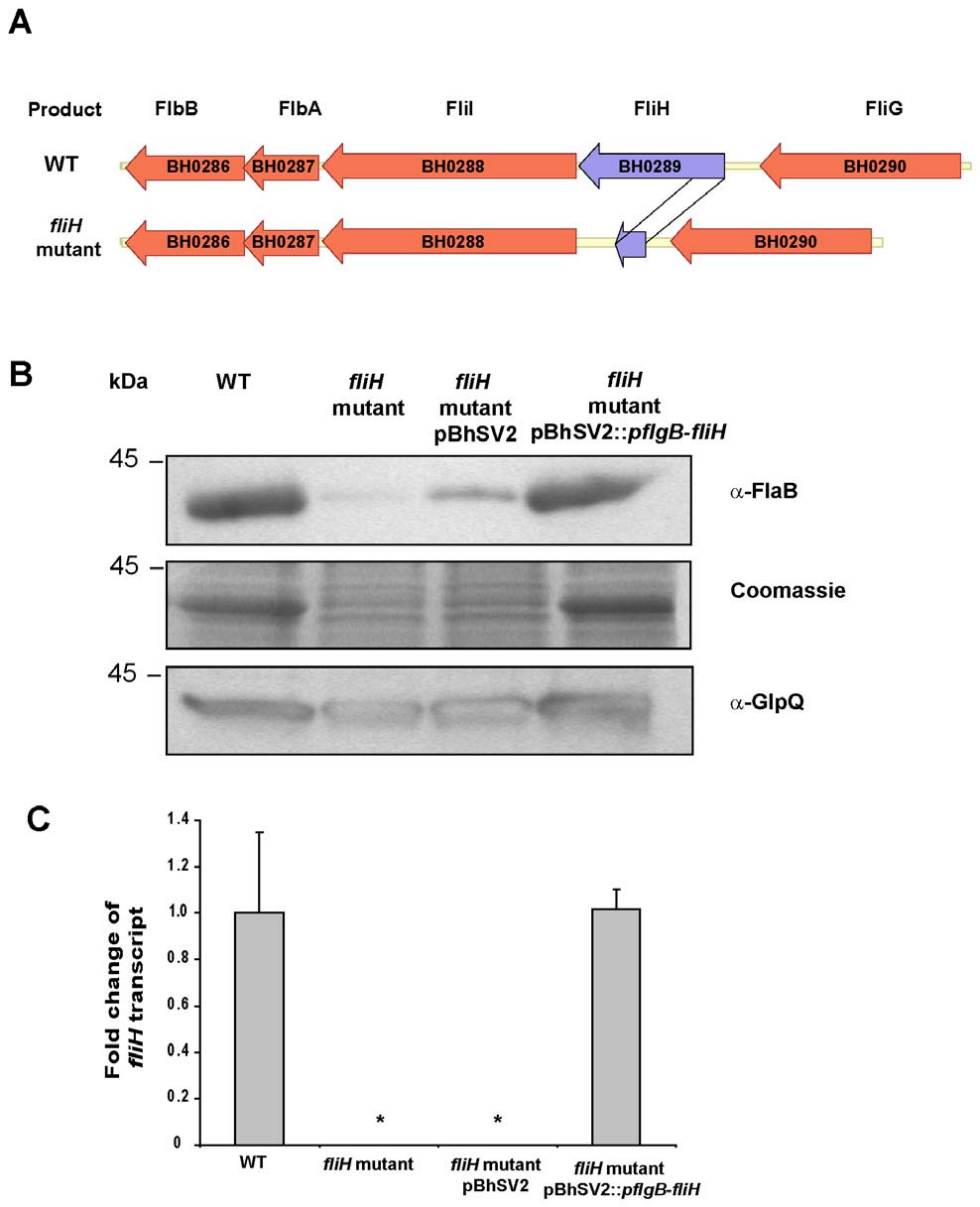
ORF diagram of the chromosomal gene *fliH* (BH0289), anti-FlaB immunoblot analysis and quantification of *fliH* mRNA using realtime-RT PCR analysis of wild-type *B. hermsii*, *fliH* mutant, *fliH* mutant pBhSV2 and complemented mutant pBhSV2::*pflgB-fliH*. (A) Schematic alignment and genetic organization of the chromosomal regions surrounding *fliH* in *B. hermsii.* (B) *B. hermsii* wild-type (WT), *fliH* mutant, *fliH* mutant transformed with the empty vector pBhSV2, and *fliH* mutant complemented with pBhSV2::*pflgB*-*fliH* analyzed by immunoblot with anti-FlaB antibody. Coomassie blue staining and anti-GlpQ immunoblot are shown to demonstrate equal loading of the cell lysates. (C) Relative amounts of *fliH* mRNA normalized to *glpQ* mRNA in the WT, *fliH* mutant, *fliH* mutant pBhSV2 and *fliH* mutant pBhSV2::*pflgB*-*fliH* as determined by qRT-PCR analyses. *, ANOVA test *P*-value <0.05 versus WT.

We first hypothesized that the lack of FlaB synthesis might be caused by a mutation in the *flaB* gene or its predicted promoter. DNA sequence analysis of this locus and the upstream flanking DNA using primers 1–4 (Table S1), revealed identical sequences in the mutant and wild-type *B. hermsii* (data not shown). This finding was consistent with the observation that some spirochetes were able to synthesize a few periplasmic flagella. Therefore, to identify the mutation causing the altered morphology and the decreased synthesis of the FlaB protein, the genome of the *B. hermsii* mutant was sequenced and compared to the chromosome of *B. hermsii* DAH (NC_010673) from which the mutant arose. Using this approach, a unique deletion of 142 bp was identified in BH0289 (*fliH*) (Fig. 4A and Fig. S1A). This deletion created a frameshift and a premature termination codon in the mutated protein (Fig. S1B) and was confirmed by DNA Sanger sequencing of a PCR-amplified fragment of this region (primers 5 & 6, Table S1). In *Salmonella enterica*, the homologue of the *B. hermsii* FliH is a cytoplasmic protein and a member of the soluble flagellar export apparatus [28]. Because of the heterogeneity in the morphology of the flagellar apparatus in the mutant spirochete, the culture was cloned again on solid medium and examined. All 12 of the new clones contained the same and identical deletion in the *fliH* gene, which suggested that the initial cloned population of the non-motile spirochete was genetically homogeneous.

In addition to the large deletion in BH0289, a codon insertion (ATT) was detected in BH0638 at the position 683830 bp of the reference chromosome of *B. hermsii* DAH (NC_010673). This mutation resulted in the insertion of a tyrosine codon at position 1283 bp of the 1335 bp BH0638 coding region near the C-terminus of a predicted Na+/H+ antiporter Nha*C*. The C-terminus of NhaC is heterogeneous among *Borrelia* species and isolates (data not shown). Two non-synonymous single-nucleotide polymorphisms (SNP) were also detected in the mutant. One (A to G) was found in BH0420 (sensory transduction protein kinase) in position 437073 bp of the reference chromosome with a predicted amino acid change of L to S. The second SNP (A to C) was identified in BH0236 (tetratricopeptide repeat family protein) in position 241144 bp of the reference chromosome, which led to a predicted amino acid change of I to R.

### Trans-complementation with *fliH* Restores FlaB Synthesis, Normal Morphology and Motility to the Mutant Spirochete

To complement the *fliH* mutant, the PCR amplified *fliH* gene was cloned into the shuttle vector pBhSV2 under the control of the *pflgB* promoter to obtain pBhSV2::*pflgB-fliH*. The *fliH* mutant was transformed with pBhSV2::*pflgB-fliH,* which restored 80% of the FlaB wild-type levels while the *fliH* mutant showed only 5% of the FlaB synthesis observed in the wild-type *B. hermsii* (Fig. 4B). The *fliH* mutant that was transformed with the empty shuttle vector pBhSV2 synthesized approximately 10% of FlaB observed in the wild-type *B. hermsii* (Fig. 4B). Equal amounts of lysate were used in our experiments, as shown by Coomassie blue staining of a SDS-PAGE acrylamide gel and anti-GlpQ immunoblot analysis (Fig. 4B). With no antibody to FliH available to confirm the protein was absent in the mutant and present in the complemented mutant, we examined the *fliH* transcript levels in the two strains. qRT-PCR analyses were performed on purified RNA using a fluorescent probe specific for BH0289. These analyses confirmed that transcription of *fliH* in the complemented mutant was restored to wild-type levels while transcripts remained undetected in the *fliH* mutant and the *fliH* mutant containing the empty shuttle vector pBhSV2 (Fig. 4C).

**Figure 5:**
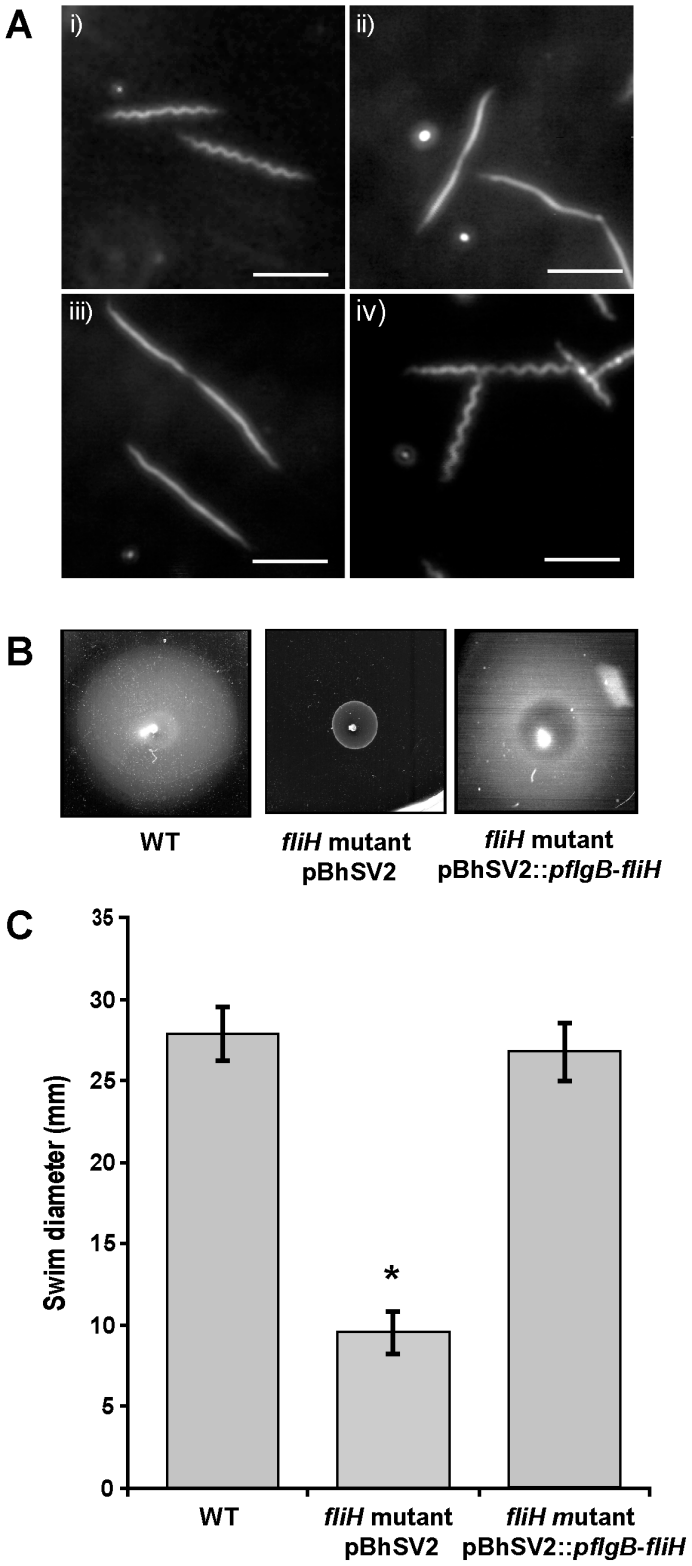
Complementation of the *fliH* mutant with pBhSV2::*pflgB*-*fliH* restores the wild-type morphology and motility. (A) Darkfield microscopy analysis of *B. hermsii* wild-type (WT) (i), *fliH* mutant (ii), *fliH* mutant transformed with empty vector pBhSV2 (iii) and *fliH* mutant complemented with pBhSV2::*pflgB*-*fliH* (iv). (B) Representative images of quantitative swimming plate assays show that the wild-type *B. hermsii* and *fliH* complemented mutant pBhSV2::*pflgB*-*fliH* swam equally whereas the *fliH* mutant swam significantly less (B & C). Results are the average of 3 independent biological experiments. The asterisk indicates that the swim diameter of the mutant was significantly less than those of the WT and complemented spirochetes (p<.05). Scale bars represent 10 µm.

Dark-field microscopy demonstrated that the complemented spirochetes were motile and their gross morphology was comparable to the wild-type *B. hermsii* DAH (Fig. 5A i and iv), while the *fliH* mutant transformed with the empty vector pBhSV2 remained straight like the *fliH* mutant (Fig. 5A ii and iii). We next examined the motility of the spirochetes with swimming motility assays. The mutant showed a markedly reduced ability to swim compared to the wild-type *B. hermsii* (Fig. 5B and C). Most importantly, swimming motility in the *fliH* mutant was restored by complementation with pBhSV2::*pflgB*-*fliH* (Fig. 5B and C). These results demonstrated that the lack of FliH caused the decreased synthesis of FlaB, reduced motility and the altered morphology observed in the *B. hermsii* mutant.

### Comparative Analyses of *flaB* Transcription

**Figure 6:**
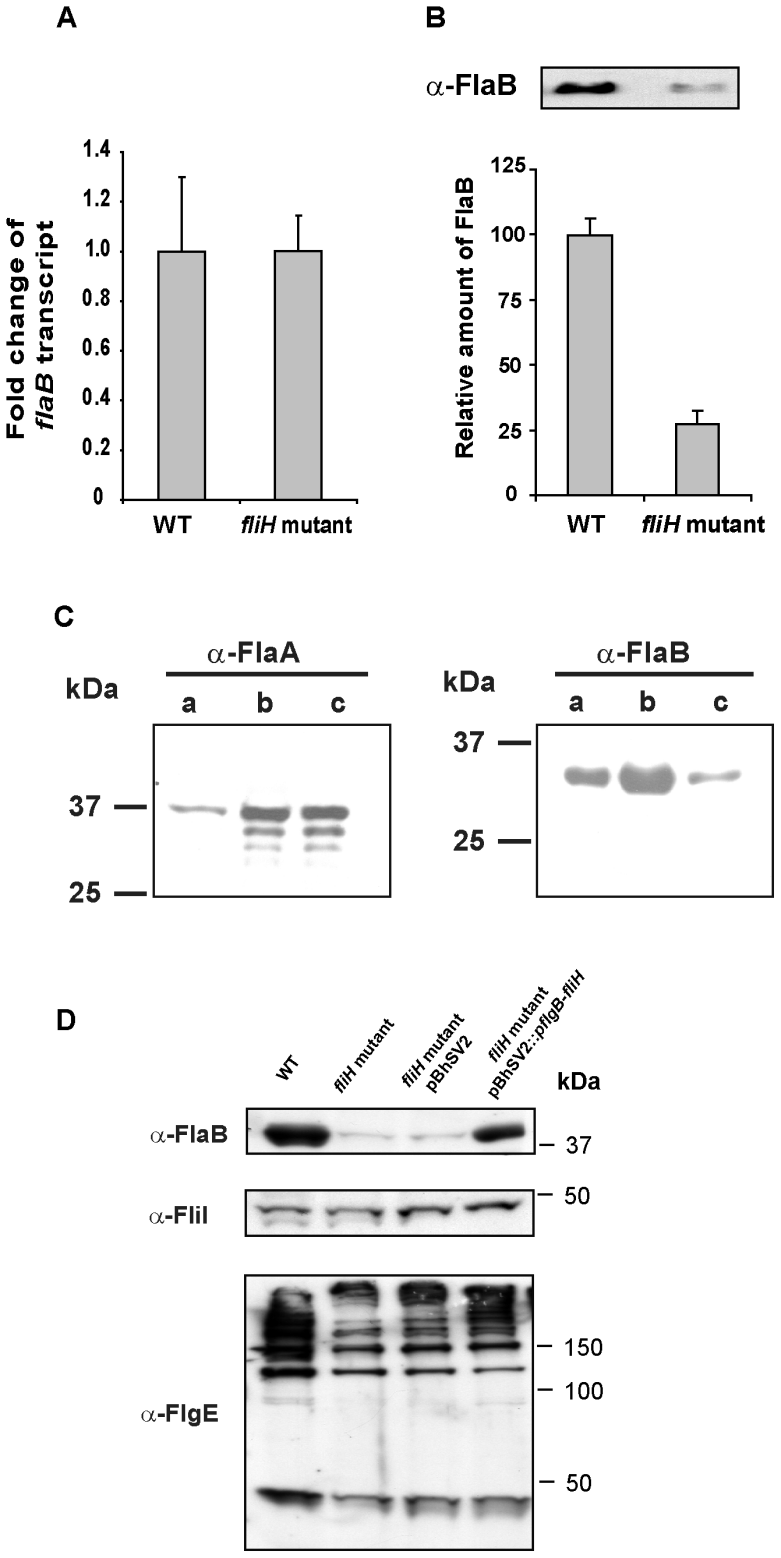
Comparison of *flaB* gene transcription and synthesis of flagellar proteins in *B. hermsii* wild-type (WT) and *fliH* mutant. (A) Fold change of *flaB* transcript normalized to *glpQ* transcript was obtained from 3 individual cultures of WT and *fliH* mutant spirochetes. (B) Relative amounts of FlaB protein were normalized to GlpQ protein measured by semi-quantitative immunoblot using 3 lysates of the WT and mutant *B. hermsii*. (C) Immunoblot analysis of purified periplasmic flagella from wild-type *B. hermsii* (lanes a), lysates of wild-type *B. hermsii* (lanes b) and the *fliH* mutant (lanes c) probed with anti-FlaA antibody (left panel) and anti- FlaB (H9724) antibody (right panel). Molecular mass standards are shown on the left in kDa. (D) Comparative anti-FlgE and anti-FliI immunoblot analyses of WT, *fliH* mutant, *fliH* mutant pBhSV2 and *fliH* mutant pBhSV2:: *pflgB-fliH* spirochetes.

To determine if the lack of FlaB was due to a transcriptional or post-transcriptional defect, the amounts of *flaB* RNA and FlaB protein in the *fliH* mutant were compared to wild-type *B. hermsii*. Experiments with spirochetes grown *in vitro* were performed in triplicate with RNA samples isolated from three independent exponential-phase cultures (5×10^7^ cells/ml) grown at 34°C. Equal amounts of *flaB* transcript were detected in the wild-type and mutant spirochetes (Fig. 6A), with no significant difference in the fold change of *flaB* transcript when normalized to the level of *glpQ* transcript. These data confirmed that the *flaB* gene was transcribed at wild-type levels in the *fliH*-deficient spirochetes. However, quantification of the FlaB protein in three independent lysates normalized against GlpQ revealed that the mutant spirochetes produced 73% less FlaB compared to the wild-type *B. hermsii* (Fig. 6B).

### Presence of FlaA, FlgE and FliI in the *fliH* Mutant

FlaA and FlaB are the two primary constituents of the periplasmic flagella. In *B. burgdorferi*, inactivation of *flaB* reduces the amount of FlaA and is controlled at the level of translation [29]. Therefore, we asked if the deficiency in FlaB in our *fliH* mutant also caused a reduction in FlaA. Conversely, a decrease in FlaA might be responsible, in part, for the decrease in FlaB. To test these hypotheses, we examined the wild-type *B. hermsii*, the mutant, and purified periplasmic flagella from wild-type cells for FlaA. The predicted molecular mass of this protein in *B. hermsii* DAH is 38,417 Da. The antibody we produced to the purified recombinant FlaA recognized a protein of this approximate size in the spirochete lysates and in the purified flagella (Fig. 6C). Interestingly, the antibody reactivity indicated that there was as much FlaA in the mutant as in the wild-type spirochetes, in spite of the greatly reduced number of flagella in the mutant spirochetes (Fig. 2). Immunoblot analysis of the same lysates and purified flagella with the anti-FlaB antibody also confirmed the presence of FlaB in the purified flagella and again demonstrated the greatly reduced amount of this protein in the FliH mutant (Fig. 6C). These results demonstrated that the reduced amount of FlaB in the *fliH* mutant was not due to a decrease in the synthesis of FlaA.

We next asked if the deficiency of FliH had an impact on the synthesis of other flagellar proteins. Comparative immunoblot analyses of lysates of *B. hermsii* DAH, the *fliH* mutant, the *fliH* mutant with pBhSV2 and the complemented *fliH* mutant with anti-FliI and anti-FlgE anti-sera showed no marked differences in synthesis of FliI (Fig. 6D). However, there was approximately 20% less FlgE in the *fliH* mutant and in the *fliH* mutant containing the empty shuttle vector pBhSV2 compared to wild-type. This moderate reduction in the amount of FlgE compared to *B. hermsii* DAH remained unchanged after transformation of the *fliH* mutant with pBhSV2::*pflgB-fliH* (Fig. 6D).

### Stability of FlaB in the *fliH* Mutant

**Figure 7:**
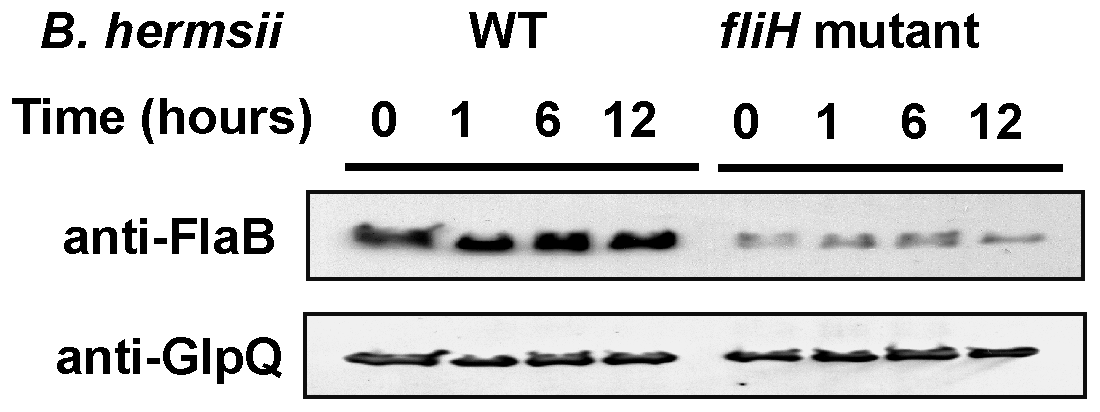
Stability of FlaB in wild-type (WT) *B. hermsii* and the *fliH* mutant. Lysates from spirochetes treated with 100 µg/ml of spectinomycin were analyzed by immunoblot using anti-FlaB and anti-GlpQ antibodies at the different time points shown.

The deficiency in the number of periplasmic flagella might be due to a post-transcriptional defect either in the synthesis of the flagella or in the assembly of the protein into axial filaments. The mutant phenotype could also be caused by a greater susceptibility of FlaB to degradation. To address this second hypothesis, we monitored the stability of FlaB with exposure tospectinomycin, which inhibits protein synthesis in borreliae [29]. Following spectinomycin treatments, lysates were analyzed by immunoblot using GlpQ as a control. For 12 hours after treatment, GlpQ was stable in both wild-type and mutant spirochetes (Fig. 7). FlaB was also stable, although semi-quantitative analyses of the bands normalized to GlpQ showed that FlaB decreased approximately 34% between 6 and 12 hr. Although the preparations varied a bit among the times sampled (Fig. 7), overall, we conclude that the deficiency of FlaB in the mutant spirochetes was not due to an increased susceptibility of the protein to degradation.

### FliH is Required for Infectivity of *B. hermsii* in Mice

**Figure 8:**
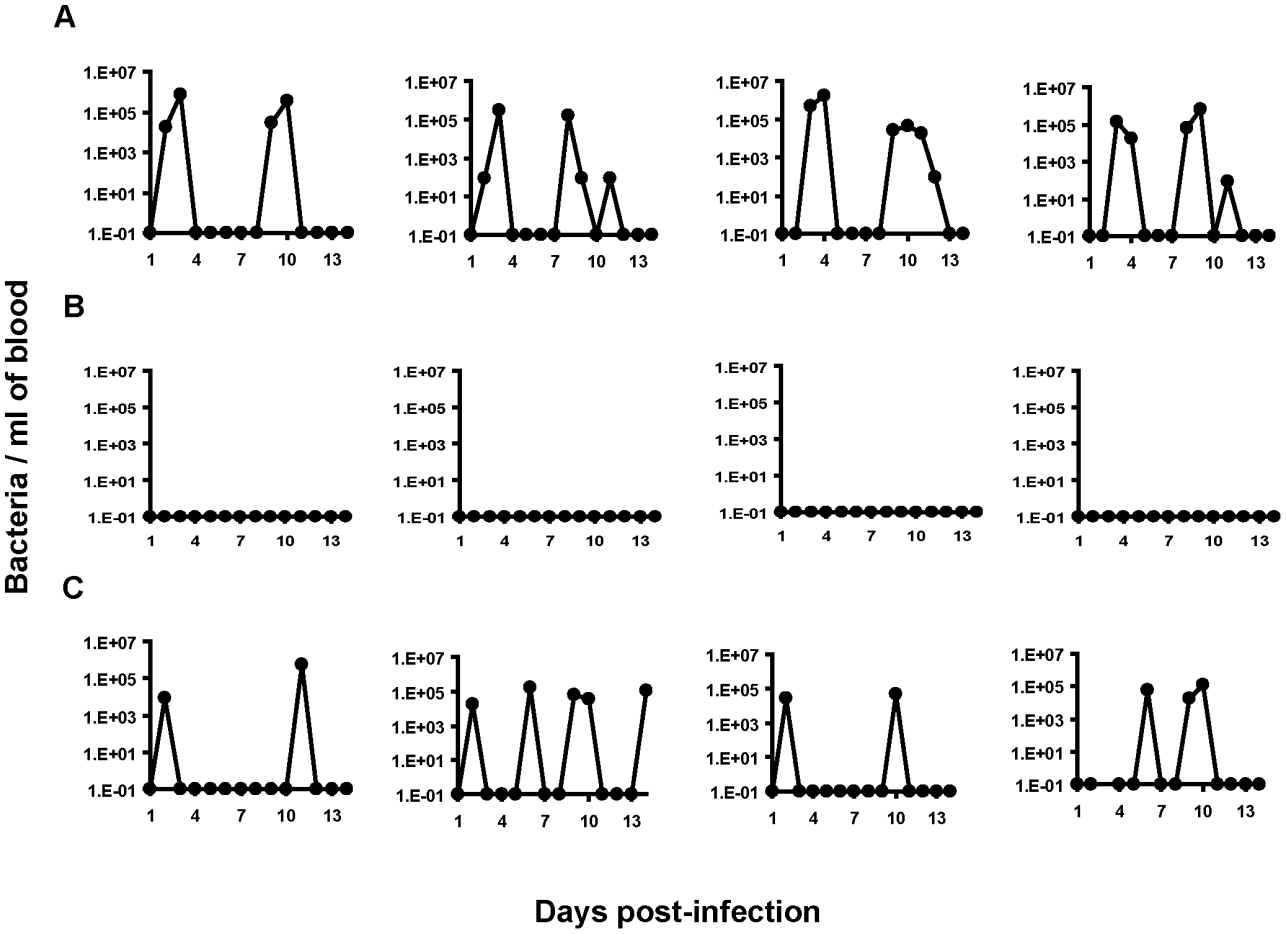
FliH is required for *B. hermsii* infectivity in mice. Kinetics of spirochetemia with wild-type *B. hermsii* (A), *B. hermsii fliH* mutant (B), *B. hermsii fliH* mutant complemented with pBhSV2::*pflgB*-*fliH* (C). Each strain was inoculated intraperitoneally into groups of 4 mice and spirochetemia was monitored daily for 14 days. Each graph represents the spirochetemia determined for one mouse. The wild-type and complemented *B. hermsii* produced primary spirochetemias and a relapse (A & C) while the FliH mutant spirochetes produced no detectable spirochetemia (B).

**Figure 9:**
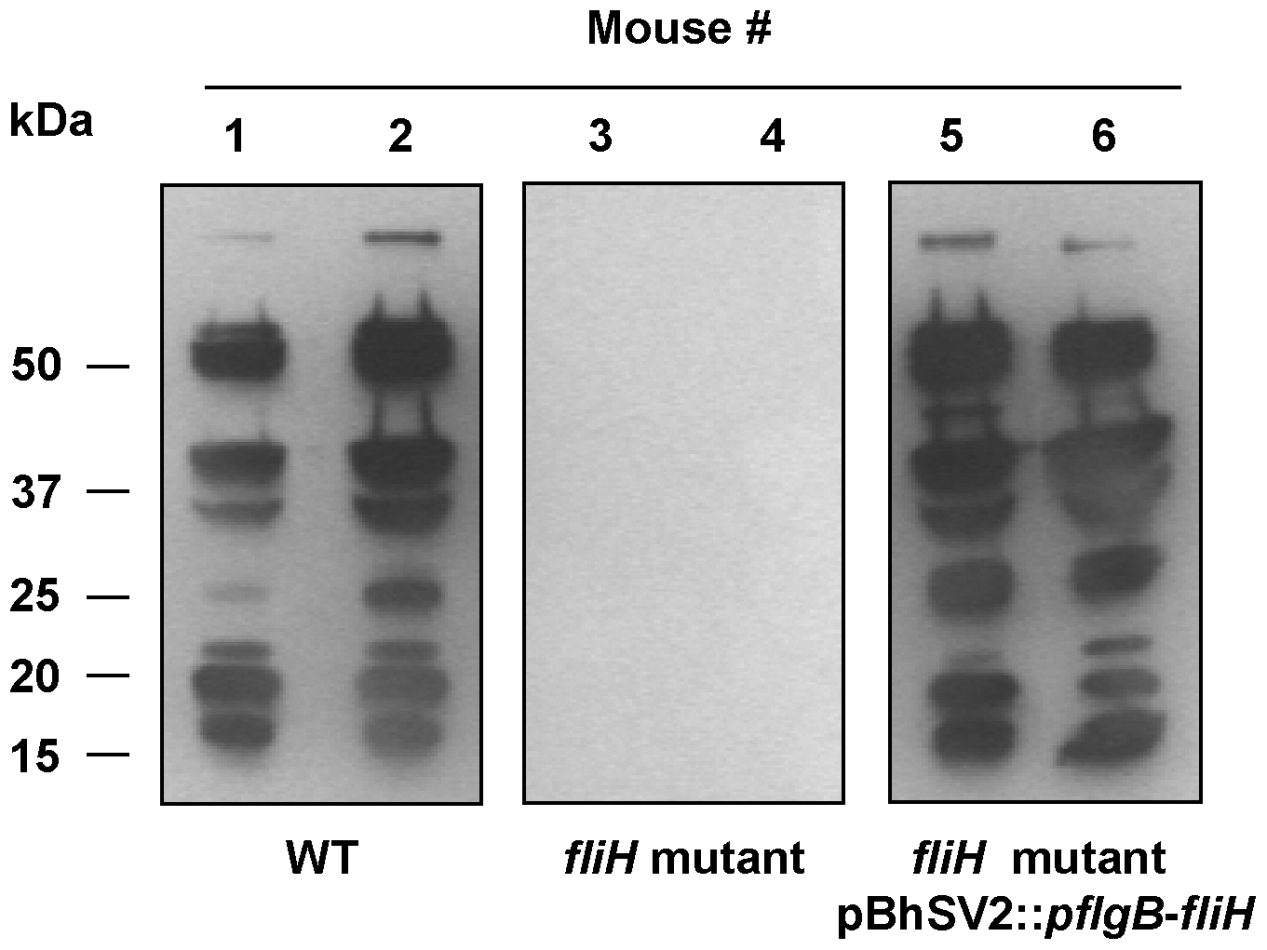
Mice inoculated with *fliH* mutant do not seroconvert to *B. hermsii*. Serum samples were examined by immunoblot for antibodies to wild-type *B. hermsii* in mice 8 weeks after inoculation with wild-type (WT) *B. hermsii* (Mice #1 and #2), the *fliH* mutant (Mice #3 and #4), and the *fliH* mutant complemented with pBhSV2::*pflgB*-*fliH* (Mice #5 and #6). Representative results are shown for two of the four mice in each group and demonstrate the complete lack of antibody detectable in the mutant-infected mice. Molecular mass standards are shown on the left in kDa.

The infectivity of the periplasmic flagella-deficient mutant was tested in mice and compared to wild-type and complemented spirochetes. All four mice injected with either the wild-type *B. hermsii* or complemented *fliH* mutant pBhSV2::*pflgB-fliH* became infected with a primary spirochetemia followed by a relapse during the 14 days after inoculation (Fig. 8A and C). In contrast, none of the four mice inoculated with the *fliH* mutant spirochetes developed a detectable spirochetemia (Fig. 8B). These results suggested that the mutation of *fliH* either greatly reduced or eliminated the ability of *B. hermsii* to survive in mice. No striking differences in the concentration of *B. hermsii* were observed in the blood of mice infected with either the wild-type *B. hermsii* (peak spirochetemia = 1.6×10^6^ bacteria/ml of blood) or the complemented mutant pBhSV2::*pflgB-fliH* (peak spirochetemia = 5.45×10^5^ bacteria/ml of blood), which confirmed that the reintroduction of a full length *fliH* in the mutant restored its virulence (Fig. 8C). Additionally, serum samples collected eight weeks after infection showed that all mice inoculated with the wild-type or complemented *fliH* mutant pBhSV2::*pflgB*-*fliH* seroconverted equally with a robust antibody response to *B. hermsii* proteins, while none of the mice injected with the *fliH* mutant showed any serological reactivity (Fig. 9). Together, these results demonstrated that FliH was essential for *B. hermsii* to infect mammals.

## Discussion

Here we describe a non-motile *fliH* mutant of *B. hermsii* that displayed a dramatic reduction in the synthesis of the FlaB protein and the number of periplasmic flagella. In contrast to other flagella-deficient mutants of spirochetes such as *Treponema denticola* or *Leptospira biflexa*, which retained their body helical shapes, the cells of the non-motile *fliH* mutant of *B. hermsii* were straight and similar to the *flaB* mutant of *B. burgdorferi* [30], [31], [32]. As demonstrated by DNA sequencing and complementation assays, this decrease of FlaB protein synthesis was directly linked to a unique genomic deletion in *fliH* (BH0289). Interestingly, the observed lack of FlaB protein was not associated with a decrease of the amount of *flaB* transcript or a major diminution of the FlaB protein stability.

The cascade of regulation that governs the expression of motility genes has been extensively described in *Escherichia coli* and *Salmonella enterica* [33], [34]. In these bacteria, the motility gene products are synthesized in a sequential manner that is coordinated by the master regulators FlhD and FlhC [35]. In *B. burgdorferi*, this transcriptional cascade control of the motility genes is lacking and many of the motility genes appear to be constitutively expressed. Transcription of motility genes is initiated by the housekeeping σ factor 70 [5], [8]. More recent studies suggest that *B. burgdorferi* may regulate the synthesis of some motility proteins at the post-transcriptional level. An insertion mutant of the *flaB* gene altered the synthesis of FlaA [29]. Depletion of the hook structural protein, FlgE, in *B. burgdorferi* induced a decreased production of FlaA and FlaB through a post-transcriptionally mediated mechanism [36]. CsrA_Bb_ down-regulates the synthesis of FlaB by inhibiting translation initiation of the *flaB* transcript [37]. In relapsing fever spirochetes, the transcriptional control of the motility genes is unknown. In a previous study, comparison of flagella loci in relapsing fever spirochetes with other *Borrelia* species showed an 85–93% DNA sequence identity to each other, which suggests that the mechanism for control of flagellin synthesis might be common to all borreliae [38]. Here we show that the *flaB* transcript level in the *fliH* mutant was comparable to wild type. Thus, the phenotypes observed in the *fliH* mutant were not likely caused by a deficiency in transcriptional regulation but rather in a post-transcriptional control system. In other bacteria, stability of flagella is partially insured by a chaperone. For example, in *S. typhimurium*, the chaperone FliS protects FliC (major flagellin protein) from degradation and controls its polymerization [35], [39]. We hypothesized that the *fliH* mutant was impaired in the synthesis of such a chaperone, which would render the FlaB protein more susceptible to degradation. However, our results showed that FlaB protein was stable in the mutant, suggesting that the phenotype of the mutant was not linked to a high susceptibility to degradation. All together, our results suggest that in *B. hermsii* the synthesis of FliH influenced the post-transcriptional processing of FlaB.

In most bacteria, export of the flagellar protein apparatus consists of six integral membrane proteins (FlhA, FlhB, FliO, FliP, FliQ, and FliR) and three soluble proteins (FliH, FliI, and FliJ) [13], [40], [41]. In concert with specific chaperones, the three soluble components are necessary for the export of all flagella substrates. FliH, FliI and FliJ form a hetero-trimer that binds to export substrates and chaperone-substrate complexes in the cytoplasm [42]. FliI is an ATPase with enzymatic activity that is necessary to drive the export process across the plane of the cytoplasmic membrane [43] and the flagellar assembly [44]. FliJ is a general chaperone preventing aggregation of export substrates in the cytoplasm [45]. Finally, FliH is a negative regulator of FliI and may prevent unnecessary ATP hydrolysis unlinked to protein translocation [40], [43]. Efficient localization of FliI at the flagellar base requires FliH [46]. Thus, a *Salmonella* species *fliH* null mutant was poorly motile and showed only a limited substrate export [43]. In *B. hermsii,* based on the morphology and few to no periplasmic flagella in the *fliH* mutant, we presume that the depletion of FliH may also lead to a reduction in export of FlaB to the periplasm and possibly to its transient accumulation in the cytoplasmic compartment.

In *B. burgdorferi*, an unidentified post-transcriptional regulator repressed FlaB synthesis when cells were mutated in the flagellar hook assembly protein FlgE [36]. Interestingly, deletion of *flgE* in *B. burgdorferi* also induced a decrease in FlaA level. In the present study, the *fliH* mutant of *B. hermsii* showed no alteration of FlaA synthesis compared to wild-type cells. This result suggests that the post-transcriptional regulators of *flaB* may differ between the two *Borrelia* species or that two regulators are able to repress the synthesis of FlaB in the *Borrelia* species.

Recently, the *B. burgdorferi* carbon storage regulator, CsrA_Bb,_ was shown to inhibit the translation initiation of the *flaB* transcript [37]. Over-expression of CsrA_Bb_ in *B. burgdorferi* reduced the mean number of periplasmic flagella. In *Bacillus subtilis*, a homologue of CsrA_Bb_ binds to FliW and this interaction governs flagellin homeostasis and a checkpoint on flagellar morphogenesis [47]. In the absence of FliW, *B. subtilis* CsrA inhibits flagellin translation. In wild-type *B. subtilis*, the transient cytoplasmic flagellin protein (Hag*)* antagonizes the binding of FliW to CsrA. Interestingly, homologues of *fliW* and *csrA_Bb_* are present in the *B. hermsii* genome (*BH0183* and *BH0184,* respectively). While additional experiments are necessary to prove this model, it is tempting to propose that in the absence of FliH, FlaB is poorly exported to the periplasm and accumulates transiently in the cytoplasm. In return, because the protein remains attached to residual cytoplasmic FlaB, FliW (or an alternate antagonist) does not bind efficiently to CsrA_Bh_ and CsrA inhibits the translation of the *flaB* transcript.

We examined the role of FliH in *B. hermsii* virulence by assessing the infectivity of the *fliH* mutant in mice. In *Clostridium* spp., *Salmonella* spp. and *Legionella pneumophila,* flagella are not involved in virulence [48], [49]. Interestingly, their flagellar proteins share sequence and structural similarities with species of *Borrelia* [17], [50]. In *L. pneumophila*, flagella make bacteria more vulnerable to the host’s innate immune response and a flagellar mutant replicates freely in macrophages [49]. In these cases where flagella are not considered to be a virulence factor, flagella are described as major ligands among the microbial associated molecular patterns (MAMPs), which are recognized by sentinel receptors like Toll-like receptor and Nod proteins [51]. In the absence of interaction between MAMPs and sentinel receptors, epithelial cells do not respond to bacterial infections. Therefore, flagellar mutants of *Salmonella* species are more virulent than the wild-type strains and they cause a more severe disease in murine models [48]. In our study, the *fliH* mutant compared to wild-type *B. hermsii* was not infectious in mice.

While the periplasmic flagella in spirochetes are not located on the outer surface of the cell and should not be exposed to the host’s humoral immunity, the axial filaments of the borrelia periplasmic flagella generally elicit an antibody response in infected patients [52]. This high antigenicity of FlaB protein suggests that some of the flagella proteins are released in their host during infection and could therefore potentially interact with sentinel receptors. Alternatively, flagella and motility are adhesion virulence factors [53]. In *Campylobacter jejuni*, *Vibrio cholerae* and *Helicobacter pylori,* motility influences host colonization by promoting migration through viscous milieus such as gastrointestinal mucus [54], [55]. A lack of periplasmic flagella in *B. burgdorferi* decreased its ability to bind to extracellular matrix components like type I collagen [56]. This decrease in binding suggested that adhesion to the extracellular matrix (ECM) could be mediated by the periplasmic flagella. Lack of infectivity of the non-motile *B. hermsii fliH* mutant might be linked to its inability to interact efficiently with the ECM. In *B. burgdorferi*, loss of periplasmic flagella observed after *in vitro* passage also correlated with lower invasiveness [57]. In addition to a decrease in its adhesion properties, invasiveness of the *fliH* mutant of this study might also be affected. Decreased invasiveness due to lack of motility would prevent dissemination in both mammals and ticks. This hypothesis agrees with findings that a *B. burgdorferi* isolate with decreased expression of flagella neither produced systemic infection or persisted in the skin of mice [58]. Most recently, Lin and coworkers described a *fliH* mutant in *B. burgdorferi* that was produced by a random transposon mutagenesis of the spirochete [59]. This mutant was nearly non-motile and “string-like” in its gross morphology, was non-infectious by tick-bite and had a reduced infectivity when inoculated by needle injection into mice.

In conclusion, we have characterized a *fliH* mutant of *B. hermsii* that is impaired in the synthesis of FlaB protein, has few or no flagella, lacks motility and is noninfectious in mice. Our data suggest that the synthesis of FlaB in *B. hermsii* is regulated at the post-transcriptional level. This regulation system is influenced by the presence of FliH, is independent of FlaA and controls directly or indirectly the number of periplasmic flagella. While our investigation did not utilize directed gene inactivation as has been described for *B. burgdorferi* [17] and *B. hermsii* [60], we believe that our analysis of the natural mutant described herein using new generation sequencing provides insights regarding the importance of FliH in the post-transcriptional regulation of flagella and in motility for infectivity of relapsing fever spirochetes in mammals.

## Materials and Methods

### Ethics Statement

The Rocky Mountain Laboratories,NIAID, NIH, Animal Care and Use Committee reviewed and approved our study protocol for infecting and sampling mice with relapsing fever spirochetes. All work in our study was conducted adhering to the institution’s guidelines for animal husbandry, and followed the guidelines and basic principals in the United States Public Health Service Policy on Humane Care and Use of Laboratory Animals, and the Guide for the Care and Use of Laboratory Animals. The *B. hermsii* used in our studies originated from a broth culture of an anonymized patient blood sample collected on August 11, 1991. Diagnostic tests for relapsing fever spirochete infections are not widely available in medical diagnostic laboratories. Therefore in August 1991, the patient’s blood sample was drawn at a hospital in Spokane, Washington, and an anonymized blood sample was sent to the Rocky Mountain Laboratories for spirochete identification. The patient gave verbal consent for blood to be collected and examined for spirochete identification. The Rocky Mountain Laboratories, NIAID, NIH, Animal Care and Use Committee was aware of and approved of the use of mice to isolate spirochetes from the blood sample. The blood sample was used only for diagnostic purposes and discarded 20 years ago with no research performed on the human sample and an IRB review and approval was not required. The culture of *B. hermsii* became an anonymized and coded sample in the RML collection of bacterial isolates.

### Borrelia Strains and Cultivation

*Borrelia hermsii* DAH were isolated after passage in laboratory mice and BSK-II medium [61]. A 10^−3^ dilution of a stationary phase culture of the spirochete, passage 5, was inoculated into the first 8 wells of two 96-well tissue culture plates and serially diluted to clone spirochetes by limiting dilution as described previously [62]. The plates were incubated in a candle jar for 2 weeks at 33°C and then all wells were examined for spirochetes by dark field microscopy. One of the final 16 wells containing growth had non-motile, straight, filamentous cells of similar size to normal relapsing fever spirochetes. The non-motile cells and typical spirochetes in another well were passaged into 15 ml tubes containing 9 ml of BSK-II medium and passaged 4 times prior to their analysis and comparison to two other isolates. *B. hermsii* HS1 (ATCC 35209) originated from *O. hermsi* collected near Spokane, Washington [63]. *B. hermsii* FRO was isolated at Rocky Mountain Laboratories in April 1987 from the blood of a human with relapsing fever in Washington [64]. *B. burgdorferi* B31 (ATCC 35210) was isolated from *Ixodes scapularis* from Shelter Island, New York [65].

### Sodium Dodecyl Sulfate-polyacrylamide (SDS-PAGE) and Immunoblotting

SDS-PAGE was performed according to Laemmli [66] with a 4% stacking gel and a 12% separating gel. After electrophoresis, gels were stained with Coomassie Brillant Blue R-250 (Invitrogen, Carlsbad, CA). Proteins in a companion gel were transferred onto a nitrocellulose membrane (Bio-Rad, Hercules, CA) for immunoblotting as described by Towbin *et al* [67]. Monoclonal antibody H9724, which recognizes all members of the genus Borrelia [68], was used at a dilution of 1∶50. Anti-GlpQ antibody SPR75 was diluted 1∶4000. Anti-FlaA polyclonal antibodies were used at a dilution of 1∶100. Anti-FliI and anti-FlgE antibodies respectively raised against FliI and FlgE of *B. burgdorferi* in the laboratory of Nyles W. Charon were used at a dilution of 1∶570 and 1∶200 respectively. Bound antibodies were detected with horseradish peroxidase-linked protein A (Zymed Laboratories, South San Francisco, CA) or [^125^I] Protein A (Amersham Biosciences, Piscataway, NJ) [69]. Proteins were detected with the SuperSignal West Pico Chemiluminescent Substrate (Thermo Scientific, Rockford, IL) according to manufacturer’s instructions. For semi-quantitative analyses, immunoblots were scanned and analyzed with ImageQuant TL software (Amersham Biosciences).

### DNA Purification

Total DNA was purified from 500 ml stationary phase cultures of the borreliae as described previously [70]. All samples were precipitated in cold 95% ethanol, washed twice in 70% ethanol, suspended in TE (10 mM Tris [pH 7.6], 1 mM EDTA), and quantified by UV at 260 nm.

### Transmission Electron Microscopy

Whole spirochete suspensions (100 µl) were adsorbed onto a 300 mesh copper grid coated with parlodion for negative staining. The supernatant was removed and the grid was washed twice in dH_2_0. The sample was stained for 30 sec with 0.5% ammonium molybdate and dried. For thin sections, pellets of spirochetes were fixed at room temperature for 1 hr in the following mixture filtered through a 0.22 µm filter: 0.4 mg 1-ethyl-3-(3dimethylaminopropyl) carbodiamide; 0.1 ml of 25% stock EM grade gluteraldehyde; 0.2 ml of 20% stock paraformaldehyde in 0.1 M NaCacodylate; 5 mg Ruthenium Red (Chroma-Gesellschaft Schmid & Co, distributed by Roboz Surgical Instrument Co., Inc., Washington, D.C.); 0.04 gm KCl; 0.1 gm CaC1_2_; 1 ml of 1 M Tricine buffer; 8.7 ml of 0.1 M sodium cacocylate. The fixed spirochetes were washed and treated with osmium and tannic acid [71], followed by 1% uranyl acetate and dehydration in 50–100% ethanol. The spirochetes were embedded in Spurr’s low viscosity resin (PolySciences, Warrington, PA) and 650 µ–750 µ sections were cut with a diamond knife. The sections were placed on grids, washed in 100% ethanol, dried, and stained with uranyl acetate and lead citrate. Whole spirochetes and sections were examined with a Hitachi Hu-11E- i electron microscope at 75 kV and photographed with Kodak SO- 163 electron image film.

### Scanning Electron Microscopy

Borrelia cultures were washed briefly with Haley’s Buffer (20 mM HEPES, 50 mM NaCl, pH 7.6) and 20 µl of the suspension was settled on silicon chips placed in a 24-cell plate for approximately 15 min and fixed with 2.5% glutaraldehyde in 0.1 M sodium cacodylate buffer. Specimens were post-fixed with 1% osmium tetroxide in dH2O, dehydrated through a graded ethanol series and critical point dried in a Bal-Tec cpd 030 drier (Balzer, Bal-Tec AG, Balzers, Liechtenstein). The specimens were then coated with 75 Å of iridium in an IBS ion beam sputter coater (South Bay Technology, Inc., San Clemente, CA) and imaged on a Hitachi SU-8000 SEM (Hitachi, Pleasanton, CA.).

### Cryo-Electron Tomography (Cryo-ET)

Aliquots (4 µl) of formalin-inactivated borrelia cultures mixed with 10 nm protein-A gold for fiducial alignment were applied to freshly glow-discharged 200 mesh Multi-A Quantifoil copper grids suspended by forceps in the FEI Mark IV Vitrobot. Specimens were vitrified after blotting for 2.5 sec with a blot force of 5 at 82% relative humidity, by plunge freezing into liquid ethane. Using a linear tilt scheme, single axis tilt series were collected on a 300 kV Titan Krios transmission electron microscope (FEI, Hillsboro, OR). Images captured over a tilt range of +/−60° (2° increments) at −3 µm defocus and total electron dose of 120 e^−/^Å^2^were recorded on a 2048×2048 pixel GIF2002 (Gatan) CCD camera using the automated tomography acquisition software (Xplore 3D, FEI). Resulting images had a pixel size of 1.03 nm. The tilt series were aligned using the IMOD software package (version 4.6.21) (University of Colorado) and SIRT reconstructions of 15 iterations were performed. All 3-D surface models were created from unfiltered tomograms with inverted contrast by manually selecting areas of interest and smoothing the 3-D volumes using the Amira Visualization Package (version 5.4.5, VSG FEI, Hillsboro, OR).Two-dimensional images were collected with the Gatan US4000 4080×4080 pixel CCD camera with a dose of approximately 100 e^−/^Å^2^ with a pixel size of 3.9 nm (Gatan Inc., Pleasanton, CA).

### Whole Genome Sequencing

A Rapid Fragment library was prepared following the manufacturer’s protocols (Roche Applied Science, Mannheim, Germany). The genomic DNA library was sequenced on the Genome Sequencer FLX (454 Life Sciences/Roche) and generated a total of 274,883 reads. The reads were mapped to the genome sequence *B. hermsii* DAH (NC_010673) using GS Reference Mapper v 2.5 (454 Life Sciences) that resulted in a 54× coverage.

### Construction of the Complementation Plasmid and Strain

The genes *fliH* and *fliI* were amplified by PCR (Promega Go-Taq, Promega, Madison, WI) with primers 7 and 8 (Table S1) from wild-type *B. hermsii* DAH genomic DNA with an initial denaturation step 95°C for 5 min followed by 35 cycles of 94°C for 30 sec, 55°C for 30 sec, 72°C for 2.5 min and 1 cycle for 7 min at 72°C. The PCR product was digested with *Nde*I and *Bam*HI and fused to the *flgB* promoter by cloning into pTABhFlgB-Kan [60] digested with *Nde*I and *Bam*HI. The resulting *pflgB*-*fliHI* fragment was amplified by PCR with primers 16 and 17 (Table S1), digested with *Ngo*MIV and *Nco*I and ligated into the shuttle vector pBhSV2 (containing *pflaB-Kan*) digested with *Xma*I and *Nco*I. To eliminate *fliI*, the plasmid was linearized with *Nco*I and amplified with primers 9 and 10 (Table S1) with the Expand Long Template PCR System (Roche) according to manufacturer’s instructions. Conditions were an initial heating at 92°C for 2 min, 11 cycles of 94°C 10 sec, 55°C for 30 sec, and 68°C for 5 min; then 35 cycles of 94°C for 10 sec, 55°C for 30 sec, 68°C for 5 min plus 20 sec/cycle, followed by a final extension of 68°C for 20 min. The amplicon was gel-purified with the Qiagen Gel Extraction Kit (Qiagen Inc., Valencia, CA), digested with *Kpn*I and re-ligated to itself. The resulting construct and the empty shuttle vector were transformed (25 µg) into the *B. hermsii* mutant and clones of the transformed spirochetes were selected with kanamycin in mBSK-c medium by limiting dilution as previously described [60].

### Swimming Assay

Spirochetes were centrifuged from the medium, washed in PBS-MgCl_2,_ and suspended in mBSK-c diluted 1∶5 in PBS-MgCl_2_ to a concentration of 2.5×10^8^ cells/ml. Four ul containing 1×10^6^ cells were inoculated into agar plates containing 30 ml mBSK-c diluted 1∶5 in PBS-MgCl_2_ with 0.35% agarose and kanamycin (200 ug/ml) when necessary. The swim diameters of the spirochetes were measured on the plates after 12 days [72].

### RNA Isolation

Total RNA was isolated from 15 ml *B. hermsii* DAH cultures (5×10^7^ cells/ml) using the RNeasy kit (Qiagen, Inc.) and contaminating DNA was removed using the RNase-free DNase Set (Qiagen, Inc.). RNA quality and integrity were assessed with the RNA 6000 Nano LabChip Kit with an Agilent 2100 Bioanalyzer (Agilent Technologies, Inc., Wilmington, DE). Concentrations of total RNA were determined with an Ultrospec 3000 UV spectrophotometer (Amersham Biosciences, Inc.).

### Realtime RT-PCR

Oligonucleotides and probes were designed with Primer Express 2.0 software (Applied Biosystems, Foster City, CA) and purchased from Applied Biosystems (Table S1, primers 11–13 and 18–23). The *glpQ* probe consisted of an oligonucleotide labeled at the 5′end with 6-carboxyfluorescein (6-FAM) as the reporter and at the 3′ end with carboxytetramethyl rhodamine (TAMRA) as the quencher. The *flaB* and *fliH* probes were labeled with the VIC reporter and the TAMRA quencher. QRT-PCR was performed with the TaqMan Gene ExpressionMaster Mix following the manufacturer’s instructions (Applied Biosystems). The RT-PCR mixture (20 µl) contained 200 nM of each gene-specific primer and 250 nM of each probe. cDNA was made from 1 µg RNA using the High Capacity cDNA RT Kit according to manufacturer’s instructions (Applied Biosystems). The cDNA was diluted to 500 µl with nuclease-free water and 5 µl were used in the reaction mix. Amplification and detection of specific products were performed with the ABI ViiA7 Real Time detection system (Applied Biosystems) with the following conditions: heating to 50°C for 2 min, then 95°C for 10 min, followed by 40 cycles at 95°C for 15 sec and 60°C for 1 min. Relative gene expression data were analyzed using the 2^−ddCt^ method and normalized to *glpQ* transcription [73].

### Analysis of the Turnover of FlaB

The turnover of FlaB in the wild-type and mutant *B. hermsii* was examined as previously described [29].The spirochetes were cultured at 34°C in BSK-H medium (Sigma-Aldrich Co., St. Louis, MO) supplemented with 12% rabbit serum. Two ml of this culture containing 5×10^8^ cells/ml were added to 40 ml of additional medium with spectinomycin (final concentration, 100 µg/ml). These cultures were then incubated at 34°C and spirochetes were examined at 0 to 12 hr by Western immunoblot with the anti-flagellin monoclonal antibody H9724 to determine the presence of FlaB. The amount of GlpQ was also monitored in the same lysates with the rabbit anti-GlpQ antibody SPR75.

### Production and Purification of His-Tag Fusion Proteins

The *flaA* gene of *B. hermsii* DAH was amplified from genomic DNA by PCR using primers 14 and 15 (Table S1). The PCR products were cloned into the pBAD202 directional TOPO-vector following the manufacturer’s instructions (Invitrogen). The resulting plasmids were transformed into *E. coli* BL21-(DE3) and colonies were selected on LB agar with ampicillin (100 µg/ml). Clones were tested for synthesis of recombinant proteins after induction with 0.2% arabinose (Sigma-Aldrich) at 30°C for 18 hr. His-tagged fusion proteins were purified under native conditions with a nickel Sepharose High Performance chromatography column according to the instructions of the manufacturer (Amersham Biosciences).

### Production of Polyclonal Antibodies

Purified recombinant FlaA protein was dialysed in phosphate-buffered saline (PBS), suspended in a final volume of 10 ml of PBS-Ribi Adjuvant R-730 (Corixa, Hamilton, MT) and used to immunize rabbits by subcutaneous and intramuscular inoculation. The rabbits received one primary immunization and three boosts at 3-week intervals. Three weeks after the third boost, blood samples were collected from the rabbits.

### Isolation of Periplasmic Flagella

Periplasmic flagella were purified as described previously with few modifications [74]. Briefly, *B. hermsii* cells (5×10^7^ cells/ml) were harvested from cultures, washed twice with PBS and suspended in 100 ml of 0.1 M Tris pH 8, 1% Triton X-100. After 1 hr of incubation at 37°C, cell cylinders were harvested and resuspended in 25 ml of 0.1 M Tris pH 8 containing 1-mm-diameter glass beads (Sigma). The suspension was then vortexed for 1 min, transferred to a new tube and centrifuged for 15 min at 15,000×g at 4°C. The supernatant was centrifuged at 100,000×g for 1 hr at 4°C and the pellet of periplasmic flagella was suspended in PBS.

### Spirochete Infections in Mice

Stationary-phase cultures of the wild-type *B. hermsii* DAH, the *fliH* mutant, and the complemented spirochete with pBhSV2::*pflgB-fliH* were each inoculated intraperitoneally into 4 adult RML Swiss-Webster laboratory mice. Each inoculum contained approximately 500 spirochetes. Blood was collected daily from the 12 mice for 14 consecutive days after inoculation by nicking the tip of the tail while the mice were anesthetized with isoflurane. A drop of blood was expressed from the tail of each mouse and 2.5 µl was transferred into a 0.375 mm diameter circle etched onto a glass microscope slide and the blood was spread evenly with a toothpick within the entire area (71.25 mm^2^). The samples were dried at RT, stained with Giemsa and examined for spirochetes by light microscopy with a Nikon Eclipse E800 microscope with a 60× oil immersion objective lens (area of one field = 0.126 mm^2^). Twenty-five microscope fields were examined for each sample, which totaled approximately 0.11 µl of blood. The number of spirochetes observed in the 25 fields was then adjusted to the number of spirochetes per ml of blood to compare the cell densities produced by the different isogenic strains of spirochetes. Serum samples from the mice were collected 8 weeks after injection and tested by immunoblot analysis as described above at a dilution of 1∶400 with the wild-type *B. hermsii* whole-cell lysate.

## Supporting Information

Figure S1 **Alignments of (A) the** ***fliH*** **genes and (B) the predicted amino acid sequences of FliH from WT** ***B. hermsii*** **and the isogenic** ***fliH*** **mutant.**
(PDF)

Table S1 Oligonucleotides used for PCR, sequencing and Realtime RT-PCR.
(DOC)
