# Supplementary material for: Periplasmic Flagellar Export Apparatus Protein, FliH, Is Involved in Post-Transcriptional Regulation of FlaB, Motility and Virulence of the Relapsing Fever Spirochete Borrelia hermsii
Source: PLoS One. 2013 Aug 29;8(8):e72550. doi: 10.1371/journal.pone.0072550 (PMC3757020; doi:10.1371/journal.pone.0072550)
Supplement: Figure S1 — Alignments of (A) the fliH genes and (B) the predicted amino acid sequences of FliH from WT B. hermsii and the isogenic fliH mutant. (PDF) [file pone.0072550.s001.pdf]

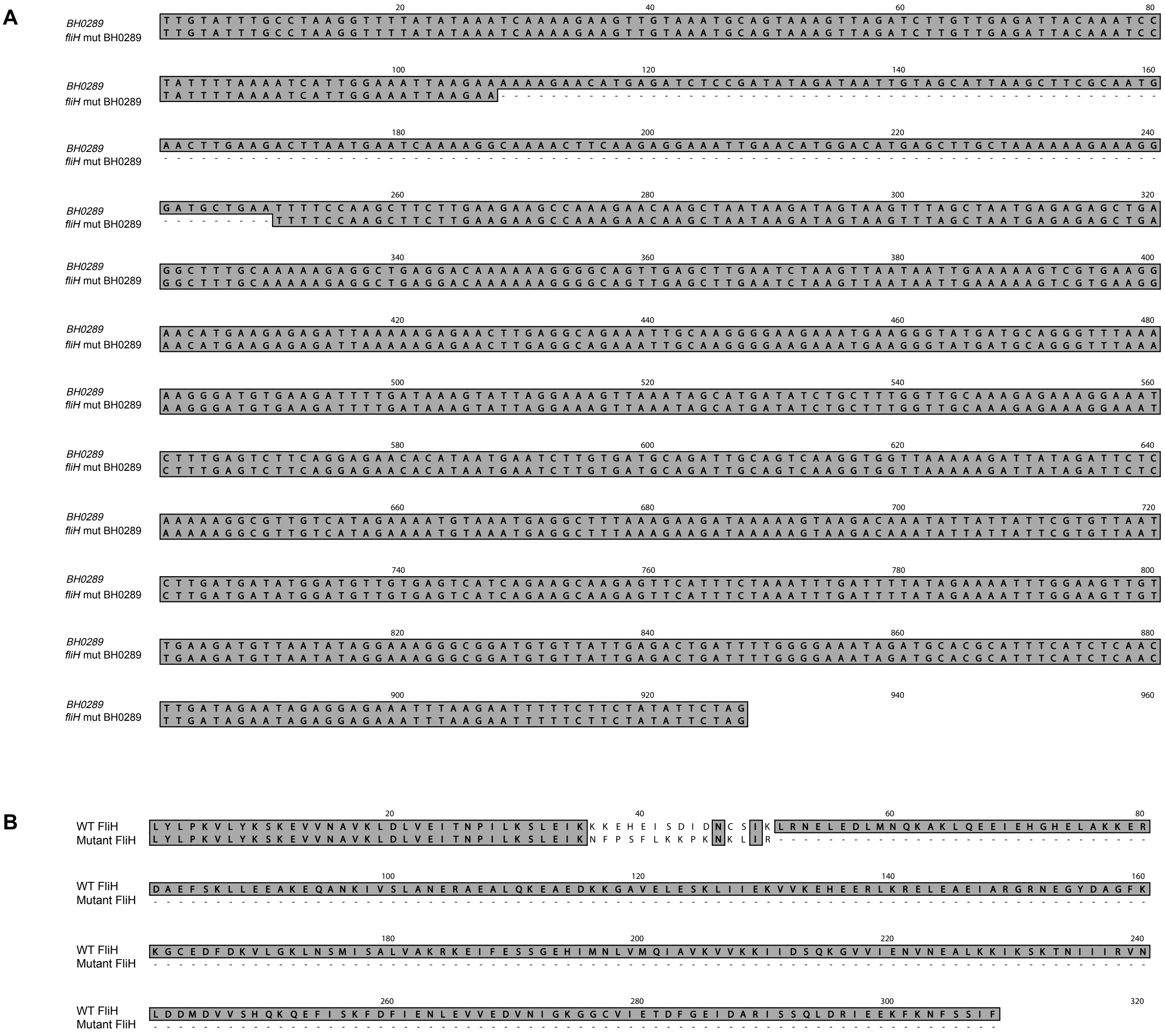
A
BH0289
fliH mut BH0289
TTGTATTTGCCTAAGGTTTTATATAAATCAAAAGAAGTTGTAAATGCAGTAAAGTTAGATCTTGTTGAGATTACAAATCC
TTGTATTTGCCTAAGGTTTTATATAAATCAAAAGAAGTTGTAAATGCAGTAAAGTTAGATCTTGTTGAGATTACAAATCC
TATTTTAAAATCATTGGAAATTAAGAAAAAGAACATGAGATCTCCGATATAGATAATTGTAGCATTAAGCTTCGCAATG
TATTTTAAAATCATTGGAAATTAAGAA--
AACTTGAAGACTTAATGAATCAAAAGGCAAAACTTCAAGAGGAAATTGAACATGGACATGAGCTTGCTAAAAAAAGAAAGG

GATGCTGAATTTTCCAAGCTTCTTGAAGAAGCCAAAGAACAAGCTAATAAGATAGTAAGTTTAGCTAATGAGAGAGCTGA
---------TTTTCCAAGCTTCTTGAAGAAGCCAAAGAACAAGCTAATAAGATAGTAAGTTTAGCTAATGAGAGAGCTGA
GGCTTTGCAAAAAGAGGCTGAGGACAAAAAAGGGGCAGTTGAGCTTGAATCTAAGTTAATAATTGAAAAAGTCGTGAAGG
GGCTTTGCAAAAAGAGGCTGAGGACAAAAAAGGGGCAGTTGAGCTTGAATCTAAGTTAATAATTGAAAAAGTCGTGAAGG
AACATGAAGAGAGATTAAAAAGAGAACTTGAGGCAGAAATTGCAAGGGGAAGAAATGAAGGGTATGATGCAGGGTTTAAA
AACATGAAGAGAGATTAAAAAGAGAACTTGAGGCAGAAATTGCAAGGGGAAGAAATGAAGGGTATGATGCAGGGTTTAAA
AAGGGATGTGAAGATTTTGATAAAGTATTAGGAAAGTTAAATAGCATGATATCTGCTTTGGTTGCAAAGAGAAAGGAAAT
AAGGGATGTGAAGATTTTGATAAAGTATTAGGAAAGTTAAATAGCATGATATCTGCTTTGGTTGCAAAGAGAAAGGAAAT
CTTTGAGTCTTCAGGAGAACACATAATGAATCTTGTGATGCAGATTGCAGTCAAGGTGGTTAAAAAGATTATAGATTCTC
CTTTGAGTCTTCAGGAGAACACATAATGAATCTTGTGATGCAGATTGCAGTCAAGGTGGTTAAAAAGATTATAGATTCTC
AAAAAGGCGTTGTCATAGAAAATGTAAATGAGGCTTTAAAGAAGATAAAAAGTAAGACAAATATTATTATTCGTGTTAAT
AAAAAGGCGTTGTCATAGAAAATGTAAATGAGGCTTTAAAGAAGATAAAAAGTAAGACAAATATTATTATTCGTGTTAAT
CTTGATGATATGGATGTTGTGAGTCATCAGAAGCAAGAGTTCATTTCTAAATTTGATTTTATAGAAAATTTGGAAGTTGT
CTTGATGATATGGATGTTGTGAGTCATCAGAAGCAAGAGTTCATTTCTAAATTTGATTTTATAGAAAATTTGGAAGTTGT
TGAAGATGTTAATATAGGAAAGGGCGGATGTGTTATTGAGACTGATTTTGGGGAAATAGATGCACGCATTTCATCTCAAC
TGAAGATGTTAATATAGGAAAGGGCGGATGTGTTATTGAGACTGATTTTGGGGAAATAGATGCACGCATTTCATCTCAAC
TTGATAGAATAGAGGAGAAATTTAAGAATTTTTCTTCTATATTCTAG
TTGATAGAATAGAGGAGAAATTTAAGAATTTTTCTTCTATATTCTAG
B
WT FliH
Mutant FliH
LYLPKVLYKSKEVVNAVKLDLVEITNPILKSLEIKKKEHEISDIDNCSIKLRNELEDLMNQKAKLQEEIEHGHELAKKER
LYLPKVLYKSKEVVNAVKLDLVEITNPILKSLEIKNFPSFLKKPKNKLIR------------------------------
DAEFSKLLEEAKEQANKIVSLANERAEALQKEAEDKKGAVELESKLIIEKVVKEHEERLKRELEAEIARGRNEGYDAGFK

KGCEDFDKVLGKLNSMISALVAKRKEIFESSGEHIMNLVMQIAVKVVKKIIDSQKGVVIENVNEALKKIKSKTNIIIRVN

LDDMDVVSHQKQEFISKFDFIENLEVVEDVNIGKGGCVIETDFGEIDARISSQLDRIEEKFKNFSSIF
