# Supplementary material for: Periplasmic Flagellar Export Apparatus Protein, FliH, Is Involved in Post-Transcriptional Regulation of FlaB, Motility and Virulence of the Relapsing Fever Spirochete Borrelia hermsii
Source: PLoS One. 2013 Aug 29;8(8):e72550. doi: 10.1371/journal.pone.0072550 (PMC3757020; doi:10.1371/journal.pone.0072550)
Supplement: Table S1 — Oligonucleotides used for PCR, sequencing and Realtime RT-PCR. (DOC) [file pone.0072550.s002.doc]

| **Code** | **Primers/probes** | **Amplified DNA/RNA** | **Sequence (5’ to 3’)** |
| --- | --- | --- | --- |
| 1 | BhflaB5' | *flaB* DNA | AATCTTTGAATTTACAGCGACAAAACAGG |
| 2 | BhflaB3' | *flaB* DNA | AAACTCCAATGCGAAAACATTACAATCC |
| 3 | Bh fla+1 | *flaB* DNA | AGAGCTTGGAATGCAACCCG |
| 4 | Bh fla-1 | *flaB* DNA | TGCCTCATCCTGATTTGCG |
| 5 | BH0289F | *fliH* DNA | GGGTGGTGAAGAAGATGTGC |
| 6 | BH0289R | *fliH* DNA | CATCATACCCTTCATTTCTTCCC |
| 7 | fliH5'-NdeI | *fliH/fliI* DNA | ATAAAGCATATGTATTTGCCTAAGGTTTTATATAAATCAAAAGAAG |
| 8 | fliI3'-NcoI | *fliH/fliI* DNA | AAGGATCCATGGTCAAGCTAATATCTCTCTCATTTCATTATCC |
| 9 | fliH3'-KpnI | pBhSV2::*pflgB-fliH* DNA | CCGGTACCACATTTTGGACCCACACTC |
| 10 | pBhSV2-KpnI | pBhSV2::*pflgB-fliH* DNA | CCGGTACCGTCAGCGTAATGCTCTGCCAGTG |
| 11 | Up-FlaB | *flaB* RNA | CAGCTAGTGATGCTGGTGTGTTAAT |
| 12 | Low-FlaB | *flaB* RNA | AAGTCAGCTGCTCAAAATGTAAAAAC |
| 13 | Probe FlaB | *flaB* RNA | TTTGCGGGTTGCATTCCAAGCTCTT |
| 14 | Up-pBADflaA | *flaA* DNA | CACCGAAGCAATTGGTGCAGG |
| 15 | Low-pBADflaA | *flaA* DNA | TTGTTTTTCAGTTCGTGCCC |
| 16 | SPM13F | *pflgB-fliHI* DNA | CGACGTTGTAAAACGACGGC |
| 17 | SPM13R | *pflgB-fliHI* DNA | GGAAACAGCTATGACCATGATTAC |
| 18 | fliH-F | *fliH* RNA | GTAGCATTAAGCTTCGCAATG |
| 19 | fliH-R | *fliH* RNA | CAAGCTCATGTCCATGTTCA |
| 20 | fliH-Probe | *fliH* RNA | CCTCTTGAAGTTTTGCCTTTTGATTCATTA |
| 21 | glpQ-F | *glpQ* RNA | TCCAGAACATACCTTAGAAGCTAAAGC |
| 22 | glpQ-R | *glpQ* RNA | TGGATCGTGCATTATAACAGGAAT |
| 23 | glpQ-Probe | *glpQ* RNA | CGCTTATGCCTTAGGAGCTGACTACCTAGAACAAG |
